# The Role of POCUS to Face COVID-19: A Narrative Review

**DOI:** 10.3390/jcm13102756

**Published:** 2024-05-07

**Authors:** Stefano Sartini, Lorenzo Ferrari, Ombretta Cutuli, Luca Castellani, Maria Luisa Cristina, Eleonora Arboscello, Marina Sartini

**Affiliations:** 1 Emergency Medicine Department, IRCCS Policlinico San Martino, Largo Rosanna Benzi 10, 16132 Genoa, Italy; ombretta.cutuli@hsanmartino.it (O.C.); caste_luca@yahoo.it (L.C.); eleonora.arboscello@hsanmartino.it (E.A.); 2 Emergency Medicine Post-Graduate School, University of Genoa, Via Balbi 5, 16126 Genoa, Italy; lorfer@outlook.it; 3 Department of Health Sciences, University of Genoa, 16132 Genoa, Italy; sartini@unige.it; 4 Hospital Hygiene, E.O. Ospedali Galliera, Via Alessandro Volta 8, 16128 Genoa, Italy

**Keywords:** COVID-19, acute respiratory failure, lung ultrasound, pneumonia, lung effusion, pulmonary embolism, deep venous thrombosis, diaphragm impairment, acute heart failure

## Abstract

COVID-19 has been a challenging outbreak to face, with millions of deaths among the globe. Acute respiratory failure due to interstitial pneumonia was the leading cause of death other than prothrombotic activation and complications. Lung ultrasound (LUS) and point-of-care ultrasound (POCUS) are widely used not only to triage, to identify, and to monitor lungs involvement but also to assess hemodynamic status and thrombotic and hemorrhagic complications, mainly in critically ill patients. POCUS has gained growing consideration due to its bedside utilization, reliability, and reproducibility even in emergency settings especially in unstable patients. In this narrative review, we aim to describe LUS and POCUS utilization in COVID-19 infection based on the literature found on this topic. We reported the LUS patterns of COVID-19 pulmonary infection, the diagnostic accuracy with respect to CT lung scan, its prognostic value, the variety of scores and protocols proposed, and the utilization of POCUS to investigate the extra-lung complications.

## 1. Introduction

Coronavirus Disease 2019 (COVID-19) is a viral infection due to a new type of coronavirus named Severe Acute Respiratory Syndrome Coronavirus 2 (SARS-CoV-2) [1]. The outbreak and the severity of the disease led clinicians to find ways to treat and manage especially acute respiratory failure (ARF) as a life-threatening condition characterized by acute onset of hypoxemia due to pneumonia caused by SARS-CoV-2 [2,3,4]. Beyond ARF, multi-organ dysfunction can be present and characterized by acute liver failure, acute kidney injury, cardiovascular disease, and thrombotic and hemorrhagic complications due to coagulation regulation impairment [5].

Over the last decades, point-of-care ultrasound (POCUS) has gained increasing consideration and widespread utilization in emergency care settings due to its availability at bed side, reliability, reproducibility, and overall cheap costs [6]. Furthermore, it is an essential tool to assess acutely ill patients in low-resource field such as prehospital settings and in those so unstable that radiological images are difficult to obtain [7,8]. 

Thus, POCUS has been found to be particularly useful as its versatility and the possibility to use it bed-side have allowed clinicians to strictly monitor compromised patients and, at the same time, to promote infection control [9]. 

The overwhelming application of POCUS in the assessment of COVID-19 patients has been reported not only for lung compromission but also to identify complications such as cardiovascular and hemodynamic compromission, thrombotic and hemorrhagic events, and diaphragm movements evaluation. Many studies has been published on the triage, diagnostic, and prognostic roles of POCUS in COVID-19 patients. 

### Objective

Due to the extensive body of literature and data on this subject, we decided to conduct this narrative review on the role of POCUS in the management of COVID-19 patients.

## 2. Materials and Methods

A comprehensive search was conducted across the PubMed/Medline, Scopus, Cochrane, and Google Scholar databases. The search incorporated the following search terms, utilizing Medical Subject Headings (MeSH) terms in adherence to the National Center for Biotechnology Information (NCBI) nomenclature and guidelines: “Lung AND ultrasound OR Point of Care Ultrasound AND COVID-19 OR SARS-CoV2”.

Inclusion criteria encompassed retrospective studies, case reports, and case series published in either English or Italian, with no temporal constraints, exclusively focusing on patients in Emergency Departments (ED) or Intensive Care Units (ICU). Publications concerning patients who had undergone surgery or had been admitted to geriatric wards were systematically excluded.

Two of the authors independently reviewed the literature. Articles were initially screened based on their titles and abstracts, employing the Rayyan platform for Systematic Review (https://www.rayyan.ai/, accessed on 22 January 2024). Subsequently, the full text of relevant research was acquired and rigorously assessed. Additionally, the references of the selected articles were scrutinized to ensure comprehensive inclusion of relevant research. Any instances of disagreement were resolved through discussion until a consensus was achieved.

The assessment of potential bias and the evaluation of study quality were conducted independently by two researchers employing distinct assessment tools tailored to the specific study design presented in the paper at hand. Any disagreements were solved by consensus.

A total of 1407 papers were initially selected, of which 1334 were excluded for not meeting the inclusion criteria. Subsequently, a state-of-the-art review was conducted to address more current matters in contrast to other combined retrospective and current approaches. This may offer new perspectives on issues or point out areas for further research.

## 3. Discussion

### 3.1. LUS and COVID-19

The increasing knowledge around and utilization of lung ultrasound (LUS) was extensively applied during the COVID-19 pandemic, allowing a global assessment of the disease in many areas:

- Triage (pneumonia/non-pneumonia) of symptomatic patients in hospital as well as in the prehospital setting.
- Diagnostic suspicion and awareness in the emergency department setting.
- Prognostic stratification and monitoring
- Treatment of intensive care unit patients with regard to ventilation and weaning.
- Monitoring the effect of therapeutic measures (antiviral or others).
- Reducing the number of health care professionals exposed during patient stratification [10].

LUS seems to be an ideal tool to assess COVID-19 disease, and since the first breakout, it has been recognized to be a useful, quick, flexible, bedside, non-radiating, and repeatable tool [11]. 

At the beginning of the pandemic, when the swab-PCR was the only way to make a COVID-19 diagnosis, ultrasound represented a triage instrument for patients admitted to the emergency department with shortness of breath and suspected COVID-19 infection [12], making their management easier and reducing time of care [13]. 

Studies conducted early during the pandemic have documented the presence of alterations of the echographic patterns and their correlation to the severity and prognosis of COVID-19 disease [14]; as much as in a specular point of view, it was employed to rule out infected patients without pulmonary lesions [15]. Many case reports described the possibility of making a quick evaluation and diagnosis by ultrasound for patient with respiratory failure due to COVID-19 infection [16,17]. 

#### LUS Patterns in COVID-19

LUS images reflect the “state of the aeration” of the lungs: the more the alveoli and interstitium are overwhelmed by exudate due to the inflammatory cascade, the more the ultrasound waves can reveal fluid and consolidative areas. 

In a well-aerated lung without inflammatory/infective processes, pulmonary parenchyma is not “accessible” to ultrasound waves. Only the interface between visceral and parietal pleura is visualized and its movements are the so called “sliding sign”. The interaction between ultrasound waves and piezoelectric crystals of the probe generates artifactual reverberation of the pleural line, the so-called A line [18]. 

When pulmonary interstitium is imbibed with fluids and inflammatory products due to COVID-19 disease progression, B lines starts to appear as vertical bright lines from the pleural line down to the bottom of the screen [19]. The more B lines are present, the more the lung is compromised, until they are not distinguishable (the so-called white lung). Thus, a B1 pattern and a B2 pattern are described as characterized by the presence of “non-coalescent” or “coalescent” B lines, respectively. The pleural line appears irregular, fragmented, and thickened [20].

**Figure 1:**
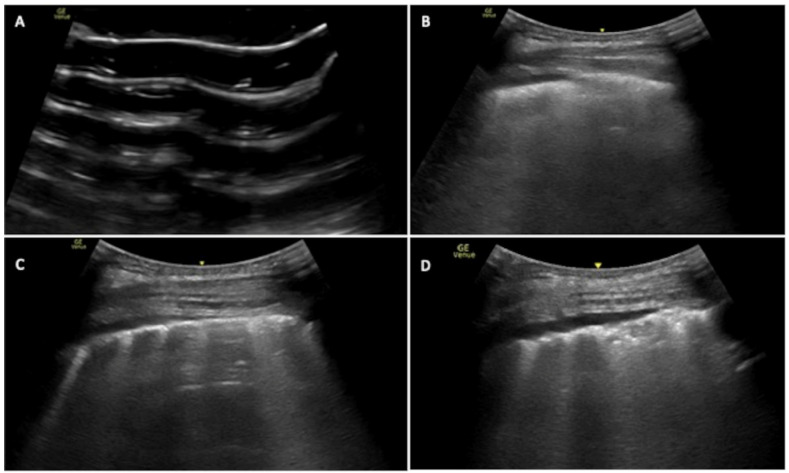
LUS patterns in COVID-19 infection: Intercostal lung scan showing the following: (**A**): pattern A = A lines are present, no B lines or consolidation; (**B**): pattern B1 = B lines are present and coalescent on the left half of the image so <50%; (**C**): pattern B2 = B lines are present >50% of the image; (**D**): pattern C = a subpleural consolidation is present surrounded by B-lines.

Another COVID-19-related finding are subpleural consolidations due to the presence of exudate in the alveolar spaces where air is no longer present. Thus, LUS can appreciate iso-hypoechoic subpleural areas that can be surrounded by B-lines: this is the so-called C-pattern (Figure 1) [21,22]. 

The most frequent LUS pathological findings in patients affected by COVID-19 pneumonia were B lines and consolidations (pattern C), respectively found in 96.9% and 83.3% [23]. In a small prospective study conducted on a group of 30 patients, B2 (multiple coalescent B-lines) and C patterns (in this study, thickening and irregularity of the pleural line and subpleural consolidations) were correlated with pathologic findings at the CT scan with a PPV of 87.1%. Those who died underwent histological sampling to evaluate alveolar damage. This revealed the presence of diffused alveolar damage and lung fibrosis, which correlated with the C pattern, while jaline membranes, organized pneumonia, and fibrosis correlated with pattern B2. This kind of histological damage did not show a correlation to patterns A (A lines or isolated B lines) or B1 (multiple well-defined B-lines) [24]. 

In another prospective observational study, LUS collected in 28 COVID-19 patients with acute respiratory failure at ICU admission and discharge was characterized by predominantly lateral and posterior non-translobar C pattern (58.9%) and B2 pattern (19.3%). Furthermore, they observed that LUS’s role in follow-up of lung lesions due to COVID-19 is not so reliable in the short term, as in this study, little difference was observed in LUS score calculation at the initial evaluation and discharge time, independently from the outcome [25].

In two prospective observational studies, the prevalence of LUS findings in COVID-19-affected patients was studied. In the first, thickening of the pleural line (>6 lung areas) were found in 49/62, confluent B lines (B2) in 38/62, separated B lines (B1) in 34/62, and consolidations (C) in 19/62 [26]. In the second, only patterns B2 and C were reported, which were found in 19.3% and 58.9% of cases, respectively [25]. 

In summary, lung POCUS is a low-cost and radiation-free method that is more feasible than CT scan, especially useful in ICUs as it does not require patients’ transport to radiology facilities and the bedside approach allows closed monitoring and appears safer in terms of infection control [27].

In patients suspected for COVID-19, lung POCUS patterns of probability combined with a medical history allowed practitioners to rule in or rule out COVID-19 pneumonia at bedside with high accuracy. In the early and mild infection stage, focal B lines are the main pattern(B1); in the progressive stage and critically ill patients, thickened pleural line, multiple or fused B-lines, and subpleural non-homogeneous consolidations are the main features (pattern B1/B2); in an advanced stage, the main features are multiple continuous large-scale hypoechoic consolidations with hepatization signs, diffused B-lines, and extensive air bronchogram signs (pattern B2/C) [28]. 

LUS’s limitations in COVID-19 are the same as in other lung diseases: lesions must be extended to the pleural surface to be detected, and consolidation has to be within an intercostal window. In the post-epidemic era, it is difficult to distinguish COVID-19 from other causes of pneumonia [27,29]. 

### 3.2. Diagnostic Accuracy: Comparison with Radiological Images

Many efforts were made during the first breakout, mainly in the emergency and intensive care unit settings, to establish a meaningful comparison between LUS and radiological images [30]. 

In this study proposed by Mongodi et al., a US self-imposed-based approach was implemented in a ICU to minimize transport for CT scan and reduce bedside CXR, comparing a year of COVID-19 management (2020) versus pre-COVID-19 (2019). They found a significant reduction in the employment of both chest CT and CXR without worsening the level of care [31]. Furthermore, in another cohort study, it was observed that monitoring of lung involvement was possible only with US instead of CT scan [32]. Conversely, different results were found in another prospective follow-up study: a lower rate of abnormalities were detected by LUS with respect to CT [26]. Furthermore, in a prospective observational study, only 67% of subjects did not present US abnormalities after 3 months from COVID-19 infection, even if the PaO_2_/FiO_2_ ratio returned to normal values (>400 mmHg), concluding that even if, during follow-up, LUS kept detecting lung abnormalities, it did not correlate with clinical state. [33]. These long-standing abnormalities were especially observed in cases affected by ARDS [34].

A retrospective cohort study conducted on 174 patients about diagnostic reliability comparing lung US with RT-PCR swab results found a sensitivity of 86%, a specificity of 71.6%, NPV of 81.7%, and PPV of 77.7%, whereas when considering only 12 complete scans, these values rose to 90.9%, 75.6%, 87.2%, and 82%, respectively. Less accurate results were found in patients with history of interstitial lung disease, severe emphysema, and chronic heart failure [35]. In another similar study on LUS diagnostic reliability, the authors observed a sensitivity of 96.9% and specificity of 91.7%, confirming previous results [36].

To remark such data, Walsh et al. found, in a retrospective study performed in symptomatic COVID-19 patients, that LUS, compared to chest CT, showed a sensitivity of 100% (95% CI 74–100), a specificity of 88% (95% CI 47–100), a positive LR of 5.8 (95% CI 1.3–25), and a negative LR of 0.05 (95% CI 0.03–0.71) in detecting lung abnormalities [37]. However, they excluded patients with chronic lung and heart diseases.

Another aspect of interest was COVID-19-induced ARDS severity evaluation. In a cohort study, a correlation was observed between the severity of ARDS and the detection of more than three B lines in the antero-superior and postero-lateral thoracic sites: it showed a sensitivity of 97% (83–100%), a specificity of 62% (50–74%), a positive predictive value of 54% (41–98%), and a negative predictive value of 98% (88–99%), with an AUC of 0.82(0.75–0.90) [38].

### 3.3. Prognostic Stratification and Monitoring

Another interesting aspect about POCUS is the evaluation of damage and clinical state evolution. There are some prospective studies about it. One of these studies simply documented that damaged lung areas reduced from the admission to the discharge time [39], although another similar study observed only a minimal variation that did not correlate with the outcome [25]. In another prospective observational study, echographic monitoring predicted the outcome. Changes in US correlated with oxygen level (Pearson = 0.591, *p* 0.033), PaO2/FiO2 ratio (Pearson = 0.167, *p* 0.008), PEEP (Pearson = 0.495, *p* 0.043), and lung compliance (Pearson = −0.572, *p* 0.016) [40]. In a similar way, other prospective studies observed a negative correlation between LUS score and PaO2/FiO2 ratio [33,41]. There are similar retrospective studies that observed LUS’s utility in monitoring patients with PaO2/FiO2 ratio <200 mmHg. Patients with a major LUS score value correlated with a worse outcome [42], or in a specular way, worse PaO2/FiO2 ratio correlated with worse LUS findings [43]. This kind of correlation was also observed for intubated patients [44]. So, LUS has also been used to evaluate responses to therapeutic acts such as non-invasive ventilation [45]; in a prospective observational study, it was observed that LUS score >12 was predictive of NIV failure [46] and it was also employed for prone positioning trial response [46,47].

Intensity of cure has been studied as a prognostic element in both prospective and retrospective studies. Worse LUS findings and higher values of LUS score correlated with worse outcome, death, and need for ICU treatment [31,43,48,49,50,51,52]. An alternative score, named PLIS, was ideated to support the decisional process, as it was correlated to SOFA score and ICU admission [53].

In a prospective longitudinal study conducted by Alharthy et al. on 100 COVID-19 patients admitted to their ICU, they found, at five to six weeks follow-up, that mortality was significantly correlated with lung alterations detected with POCUS at hospital admission [39].

### 3.4. POCUS and COVID-19-Related Issues Hemodynamic, Thrombotic and Hemorrhagic Assessment

Beyond lung damage identification, POCUS was employed for differential diagnosis, hemodynamics impairments, and thrombotic and hemorrhagic COVID-19-related complications. Extensive evidence has been produced, mainly on the utilization of POCUS to assess heart function and to investigate the presence of deep venous thrombosis. Furthermore, other applications were experienced and reported.

#### 3.4.1. Cardiovascular and Hemodynamic Assessment

The most frequent cardiac abnormality found in COVD patients was right ventricular (RV) dysfunction related to increased pulmonary circulation resistance as a result of small vessels thrombosis and hypoxemia. The reflection on the heart is increased pulmonary arterial pressure, RV afterload, RV dilatation, and dysfunction [54]. In addition, the detection of RV enables the assessment of RV-pulmonary arterial coupling and the evaluation of RV adaptability to pressure loading, which can guide clinical treatment [55]. 

Moreover, ventilatory therapy (both non-invasive and invasive) affects RV dimension and function by influencing heart–lung interactions increasing intra-thoracic pressure. 

Specific findings to be measured are as follows:

RV dimensions: dilatation is defined by a diameter > 41 mm at the base and > 35 mm at the mid-level.

RV wall thickness: RV hypertrophy is identified by thickness more than 5 mm.

Systolic pulmonary arterial pressure: RV right atrial pressure gradient can be estimated in the presence of tricuspid regurgitation by measuring the peak regurgitant jet velocity; it is important during ventilation, as afterload is augmented by positive pressure applied, to monitor pulmonary arterial pressure by means of echocardiography to detect right ventricle dilatation and/dysfunction early.

Inferior vena cava (IVC) diameter and collapse: diameter > 2.1 cm that collapses < 50% with a sniff suggests a high RA pressure of 15 mm Hg.

Tricuspid lateral annular motion (TAPSE): good correlation with RV fractional

left ventricular (LV) ejection fraction.

ACP is defined as the association of RV dilatation with a paradoxical septal motion at end-systole [21,56,57]. 

In a retrospective observational study by Doi et al., the authors found that patients with echographic signs of RV involvement (specifically the McConnell’s sign), left ventricular wall motion abnormalities, and higher D-dimer level were associated with a worse outcome [58]. 

Moreover, LV contractility assessment allowed researchers to identify cases complicated by myocarditis and myocardial infarction by looking at the LV kinetic and ejection fraction [28,59]. 

The combination of LUS and echocardiography has had a great impact on treatment decisions [54,59]. There are many case reports that report cardiac involvement in COVID-19-affected patients [60].

#### 3.4.2. Thrombotic and Hemorrhagic Assessment

Many case reports papers have described a wide employment of POCUS for thrombotic pathology identification. Also, hemorrhagic complications have been described as the identification of subarachnoid hemorrhage using trans-cranial color-coded duplex sonography added to usual POCUS [61]. 

POCUS is a reliable tool to detect thrombotic events, a common complication in COVID-19 syndrome, especially in the first breakout. Even if screening of deep venous thrombosis (DVT) was not routinely recommended, during COVID-19 infection, D-dimer conventional values were a less reliable marker to assess thromboembolism as higher values were found irrespective of its presence [62]. Thus, an extensive use of POCUS was implemented, especially in those with sever hemodynamic worsening [63]. A case report reported how the BLUE protocol was integrated with a focused echocardiography to permit a stronger diagnosis in suspected pulmonary embolism in a case of COVID-19 with a normal lung ultrasound [64]. In a multicentric prospective study by Pieralli et al., they screened for DVT with POCUS in non-ICU settings in 227 patients with COVID-19 infection. They found an overall incidence of 13.7%, older patients, and those with higher D-dimer peak (>2000 ng/mL) were more affected [65]. 

Another application of POCUS was diaphragm ultrasound (DUS) evaluation in COVID-19 patients with respiratory distress. DUS enables practitioners to obtain information regarding breathing mechanics, lung compliance, and diaphragm dysfunction [13]. Diaphragm thickening fraction (DTF), a measurement of the difference in end-inspiratory and end-expiratory diaphragmatic thickness, expressed as a percentage and diaphragm excursion (DE), the diaphragmatic altitude difference between expiration and inspiration, can be assessed with ultrasound. DTF was used to assess COVID-19 patients with severe respiratory failure on non-invasive ventilation support; if altered, it showed a correlation with the need of invasive mechanical ventilation [66]. Furthermore, DTF was used during weaning from mechanical ventilation in COVID-19 patients and was significantly correlated with pH, PaO_2_/FiO_2_ ratio, and hospital and ICU length of stay [67]. Moreover, diaphragmatic US disfunction was found in 13/132 patients affected by severe COVID-19 pneumonia at 6 months follow-up [68].

Finally, a peculiar utilization of POCUS has been reported: in a case report, it was used to detect and monitor complications as thrombotic events and subarachnoid hemorrhage in COVID-19 patients, adding transcranial color-coded duplex sonography to standard US after CT scan diagnosis [62].

### 3.5. Scores and Protocols Proposed on LUS and POCUS

The LUS score was proposed in an attempt to standardize lung US examination and interpretation. The LUS score is based on a semiquantitative score representing the lung aeration loss caused by the progression of the pathological lung parenchymal involvement. It is calculated by the sum of points from 0–3 given to 12 regions of LUS (upper and lower region of anterior, later and posterior lung regions). With the probe positioned in the intercostal space allowing full pleural visualization, points are given as follows:

0 points—presence of lung sliding with A lines or one or two isolated B lines;

1 point—moderate loss of lung aeration with three or four B lines involving <50% of each ultrasound image (septal rockets);

2 points—severe loss of lung aeration with five or more B lines involving >50% of each ultrasound image (glass rockets);

3 points—presence of a hypoechoic poorly defined tissue characterized by complete loss of lung aeration (consolidation) [69].

In a cohort study, the correlation between LUS score values and parenchymal damage extension detectable on the CT scan was measured. A LUS score mean of 15 ± 6.7 correlated with <50% involvement at CT scan, and a LUS score mean of 21 ± 6 with >50%, *p* < 0.001 [30]. Furthermore, LUS score was tested during patient monitoring, and correlation between CT scan and LUS score was maintained [70]. 

In an observational pilot study by Baciarello et al., LUS score correlation with the stage of respiratory failure in terms of PaO_2_/FiO_2_ ratio and mortality were evaluated: a cut-off of >11 showed an AUC of 0.83 with a PPV of 98% for ARF and a significant correlation with in-hospital mortality [42]. Similar results were found in a cross-sectional study in which LUS score showed a correlation with PaO_2_/FiO_2_ ratio of −0.52 (*p* < 0.001) at the multivariate analysis [71].

LUS score has been used as a value to monitor ARDS evolution and the effects of mechanical ventilation on lung aeration. Data regarding diagnostic performance of LUS score demonstrated high accuracy (93.3%), sensitivity (100.0%), and specificity (92.9%) for severe lung lesions, confirming its usefulness in the diagnosis and monitoring of critical COVID-19 patients [72].

In another retrospective study, a severity score was calculated based on LUS findings that showed good discriminatory performance to predict all-cause inpatient mortality, death or critical care admission, and escalated oxygen requirements [48].

Thus, we can conclude that LUS score is a useful tool for clinicians to assess prognosis and to monitor lung parenchymal impairment in a standardized way. 

Manivel et al. proposed the CLUE protocol which combines LUS score and the amount of oxygen requirement at the time of examination to help physicians in making disposition decisions. Patients were classified as mild, moderate, and severe risk based on LUS scores of 1–5, 6–15, and >15, respectively, plus the need for oxygen supply to obtain an adequate saturation levels. They advised admitting patients to a standard ward if at moderate risk plus oxygen supply or severe risk without oxygen supply and to ICU if at severe risk plus oxygen supply [73]. In a prospective study based on the implementation of CLUE protocol, based on regression analysis, a LUS score higher than 10 was found as an independent risk factor for intensive care requirement, a score lower than 3 for discharge, and a score over 11 for mortality [74].

Orosz et al. applied the so-called BLUE-LUSS protocol to mechanically ventilated critically ill patients. It consists of the sum of LUS score for four scans for each hemithorax (a superior point below the middle-clavicular point, a diaphragm point at the lung–liver or lung–spleen junction at mid-axillary line, an M point is at the midpoint between these two points and a posterolateral point at the intersection of posterior axillary line and the vertical line from M point). They found a significant correlation between LUS score and PaO2/FiO2 ratio but not with the inflammatory biomarkers [75]. Xue et al. evaluated the interobserver variability and correlation of disease severity of M-BLUE protocol in ICU patients. They found a good agreement between ICU practitioners and radiologists with an ICC of 0.61 and 0.60 [76].

In a prospective observational study, Torre-Macho et al. analyzed the prediction accuracy of serial LUS examinations. Their protocol was based on 10 scanned zones: each intercostal space of the upper and lower parts of the anterior, lateral, and posterior regions of the left and right chest wall were examined (four anterior and six posterior scans). For each of the 10 zones, a score from 0 to 5 was given depending on sonographic findings: A pattern (0 points), isolated B-lines defined as less than three in a 3 s clip (1 point), more than three B lines during a 3 s clip (2 points), coalescent B-lines (3 points), small (<1 cm) subpleural consolidations (4 points), and consolidations >1 cm (5 points). The total score was calculated by summing the scores of all 10 zones (range of possible scores: 0–50). The score was calculated at 0–48 h and 72–96 h from hospital admission in a total of 469 patients, with death or the need for invasive mechanical ventilation as primary endpoints. They found an AUC of 0.72 (0.58–0.85) at the second examination but an higher discrimination power for the differential score between the second and the first examination, with an AUC of 0.78 (0.66–0.90) for the primary endpoints [77].

Even if many scores and protocols have been proposed, the interpretation and standardization of the ultrasound lung images with the LUS score seems the most reliable and simple way to assess lung involvement in COVID-19 patients. 

## 4. COVUS and ORACLE Protocol

During the outbreak, many structured protocols were reported to standardize and to optimize time of examination and complications detection of COVID-19-affected patients. ORACLE and COVUS are two proposed protocols aimed to detect cardio-thoracic complications using a single sector probe. These protocols are slightly different in execution and results, as they have been ideated and are applied to establish the diagnosis of multiple associated pathologies that could influence the medical management.

**Figure 2:**
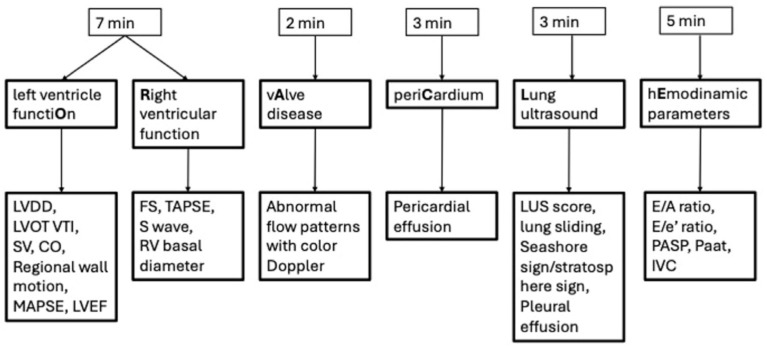
The ORACLE protocol flowchart.

**Figure 3:**
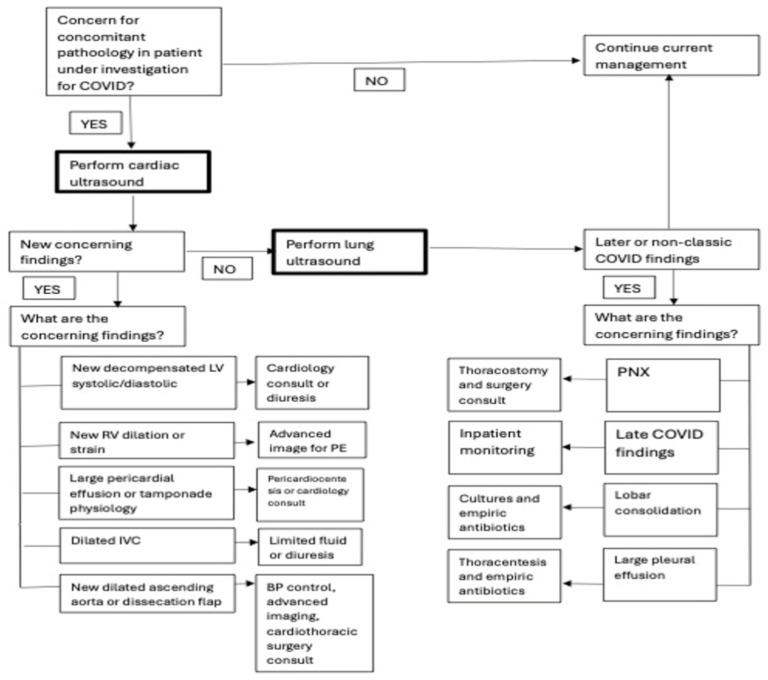
The COVUS protocol flowchart.

As reported in the respective flow charts, both protocols are designed for a cardiac-first approach, to detect life threatening conditions, and LUS integration, to identify lung complications like pleural effusion and pneumothorax (Figure 2 and Figure 3).

ORACLE can be executed in 20 min and consists of six parts (as reported in the flow chart Figure 3) that, respectively analyze left ventricle function, right ventricle function, valvular disease, pericardium, lung ultrasound (executed on six segments in each hemithorax), and hemodynamic parameters. 

COVUS divides cardiac and lung examinations. For lung examination, the lawnmower technique is suggested (instead of 12 segments examination). 

In a cross-sectional study, ORACLE protocol was applied. It permitted the authors to characterize the lung–cardiac involvement and hemodynamic status of critically ill patients, helping in the decision-making about management and treatment [78,79]. 

## 5. Limitations

POCUS application in clinical practice and its effectiveness in reducing unfavorable outcomes are affected by some limitations from many different points of views.

Availability and settings: There is a lack of ultrasound machines in specific settings like pre-hospital or in limited-resource countries; furthermore, updated software and probes are needed to obtain more reliable images. Moreover, in case of intensive use from patient to patient and a lack of disinfection and cleanliness, the probes could be a vector of infection [80].

Technical impairment: “Air” in itself is a limitation to ultrasonic waves’ propagation, and their interaction with body tissue and fluids generates artifacts that have to be recognized and correctly interpreted. Furthermore, the correct use of the different probes and the many settings allowed by the new ultrasound machine is mandatory to properly set up adequate images. Finally, the lack of standardization with specific protocols for upper and lower airway POCUS execution may limit replication and increase interobserver variability [81]. 

Competences: Education in POCUS techniques and an adequate level of experience are cardinal points to obtain a reliable POCUS assessment. Continued US utilization in daily clinical practice, comparison with other gold standard imaging exams, and support of senior team members are needed to avoid clinical errors and to improve personal skills. Specific ultrasound training program should be implemented in trainee core curriculum [82].

Scientific level evidence: Most of the published data about POCUS clinical utilization and effectiveness are based on observational study. However, it is difficult to plan studies with a strong level of evidence such as TRIAL or prospective multicentric and interventional study due to organizational and methodological impairment such as different ultrasound machines in different settings, interobserver variability, availability of ultrasonologist with the same level of competences, and contradictory outcomes identification and measurements.

Even in light of such considerations, POCUS is undoubtedly a useful clinical tool; further and stronger evidence is needed to fully support its utilization.

## 6. Conclusions

LUS and other POCUS applications in COVID-19 had a great impact on clinical management in terms of lung involvement and monitoring, identification of thrombotic and hemorrhagic complications, prognostic assessment, and infection control safety. Among clinicians, POCUS spread during the COVID-19 outbreak, and its utilization has grown in daily life, even after the infective emergency. Nowadays, POCUS can be considered a precious tool in the management of critically ill patients. Perspectives should focus on implementing LUS and POCUS utilization in daily practice and the possibility to create clinical both therapeutic and organizational pathways. This may allow researchers to plan prospective study such as trials with and without LUS utilization in the management of COVID-19 patients, looking at the real impact on clinical outcomes. 

## Data Availability

Not applicable.
